# The impact of exchanging the light and heavy chains on the structures of bovine ultralong antibodies

**DOI:** 10.1107/S2053230X2400606X

**Published:** 2024-07-01

**Authors:** John D. Clarke, Alice Douangamath, Halina Mikolajek, Marie Bonnet-Di Placido, Jingshan Ren, Elizabeth E. Fry, Dave I. Stuart, John A. Hammond, Raymond J. Owens

**Affiliations:** a The Division of Structural Biology University of Oxford Roosevelt Drive OxfordOX3 7BN United Kingdom; b Macromolecular Crystallography Diamond Light Source Harwell Science and Innovation Campus DidcotOX11 0DE United Kingdom; c Immunogenetics The Pirbright Institute Ash Road Pirbright WokingGU24 0NF United Kingdom; d Crystallography The Research Complex at Harwell Harwell Science and Innovation Campus DidcotOX11 0FA United Kingdom; e Structural Biology, The Rosalind Franklin Institute, Harwell Science and Innovation Campus, DidcotOX11 0QX, United Kingdom; The Scripps Research Institute, USA

**Keywords:** ultralong, immunoglobulin, antibodies, chain exchange, CDR3H, CDR3L, X-ray crystallography, structural immunology

## Abstract

The third complementary-determining regions of the heavy-chain (CDR3H) variable regions (VH) of some cattle antibodies are highly extended, consisting of 48 or more residues. These ‘ultralong’ CDR3Hs form β-ribbon stalks that protrude from the surface of the antibody with a disulfide cross-linked knob region at their apex that dominates antigen interactions over the other CDR loops. The structure of the Fab fragment of a naturally paired bovine ultralong antibody (D08), identified by single B-cell sequencing, has been determined to 1.6 Å resolution. By swapping the D08 native light chain with that of an unrelated antigen-unknown ultralong antibody, it is shown that interactions between the CDR3s of the variable domains potentially affect the fine positioning of the ultralong CDR3H; however, comparison with other crystallo­graphic structures shows that crystalline packing is also a major contributor. It is concluded that, on balance, the exact positioning of ultralong CDR3H loops is most likely to be due to the constraints of crystal packing.

## Abstract 2

Complementary-determining region 3 (CDR3) of the light chain interacts with the extended CDR3 of the paired heavy chain in bovine ultralong antibodies. Exchanging light chains between two different ultralong antibodies induced a small axial twist in the CDR3H β-ribbon stalk region; however, comparative crystallographic interface analysis indicates that the ultralong loops have flexibility and their positioning is affected by crystal packing.

## 1. Introduction

Cattle present a broad trimodal distribution of loop lengths of complementarity-determining region 3 on the heavy chain (CDR3H; Saini & Kaushik, 2002 ▸), with approximately 10% harbouring an ‘ultralong’ CDR3H of greater than 47 residues (Saini *et al.*, 1999 ▸; Saini & Kaushik, 2002 ▸). The ultralong CDR3H is structured as a miniature cysteine-knot domain (referred to as the ‘knob’ domain) positioned distal to the antibody and separated by a two-pass antiparallel β-stalk (Wang *et al.*, 2013 ▸; Stanfield *et al.*, 2016 ▸; Dong *et al.*, 2019 ▸). The ultralong knob domain appears to interact exclusively with the antigen without contributions from other CDRs (Liu *et al.*, 2015 ▸; Stanfield *et al.*, 2020 ▸; He *et al.*, 2021 ▸), and can even recognize the antigen in the absence of the residual antibody scaffold (Svilenov *et al.*, 2021 ▸; Macpherson *et al.*, 2021 ▸; Huang *et al.*, 2023 ▸).

Ultralong heavy chains are ubiquitously observed to pair with bovine light chains encoded by IgLV1-47 (Saini *et al.*, 1999 ▸) or its close homolog IgLV1-67 (Saini *et al.*, 2003 ▸), and these light chains show very limited sequence variation (Sinclair *et al.*, 1995 ▸). IgLV1-47 provides a relatively featureless compact CDR3L (Wang *et al.*, 2013 ▸; Stanfield *et al.*, 2016 ▸; Dong *et al.*, 2019 ▸) that accommodates and tightly interacts with ultralong CDR3H stalk regions, whereas no ultralong structures have yet been reported with a light chain encoded by IgLV1-67. Light-chain conservation within the ultralong antibody context has even allowed the engineering of functional bispecific ultralong antibodies using a scaffold with a common light chain (Klewinghaus *et al.*, 2022 ▸).

Previous work (Ren *et al.*, 2019 ▸) has shown that the pairing of bovine heavy (H) and light (L) chains can radically alter the conformation and associated binding affinities of a non-ultralong bovine CDR3H; thereby showing the potential to alter the CDR3H structure and function through light-chain contributions. In the context of limited light-chain involvement in ultralong antigen interaction, yet knowledge that CDR3Hs are affected by the light chain, this study investigates the contribution of the CDR3L sequence to the CDR3H conformation by comparing the crystallographic structures of two antigen-unknown (discovered from an adult animal treated with typical veterinary vaccines as a calf) ultralong antibodies, D08 (Valdez *et al.*, 2023 ▸) and BLV5B8 (Saini *et al.*, 1999 ▸; Wang *et al.*, 2013 ▸), with those of synthetic antibodies produced by recombinant chain-exchanged variants.

## 2. Materials and methods

### 2.1. Macromolecule production

The discovery of bovine immunoglobulin sequences is documented elsewhere, following ‘Strategy 1’ (Valdez *et al.*, 2023 ▸). Briefly, bovine immunoglobulin variable-domain forward primers and IgM, IgG and Igλ reverse primers were designed using the Bovine IgH 202 BAC assembly (Ma *et al.*, 2016 ▸). Bovine antibody VH and VL chains were amplified from sorted single B cells by semi-nested PCR using a OneStep RT-PCR Kit (Qiagen) and sequenced by Sanger sequencing (Eurofins), resulting in the discovery of the ultralong antibody D08. The heavy and light chains of the previously reported ultralong antibody BLV5B8 (Saini *et al.*, 1999 ▸; Wang *et al.*, 2013 ▸) were also selected for study. Sequence alignments are provided as supplementary figures.

Synthetic genes encoding the variable regions of the antigen-unknown D08 ultralong bovine antibody (VH and VL) were purchased from IDT Technology as gBlocks and inserted into the pOPINBOVH and pOPINBOVL expression vectors, respectively, by InFusion cloning (Ren *et al.*, 2019 ▸; Table 1 ▸). All vectors were sequenced to confirm that the clones were correct. Recombinant Fabs D08, D08* (comprising the D08 heavy chain and the BLV5B8 light chain) and BLV5B8* (comprising the BLV5B8 heavy chain and the D08 light chain) were produced by co-transfection of VH and VL vectors into Expi293 cells according to the manufacturer’s protocol (Invitrogen). Proteins were purified from culture supernatants by a combination of immobilized metal-affinity and size-exclusion chromatography using an automated protocol implemented on an ÄKTAxpress (GE Healthcare; Nettleship *et al.*, 2009 ▸). Eluted fractions were characterized by SDS–PAGE and those of interest were spin-concentrated using Amicon Ultracel-30 centrifugal filters (Merck).

### 2.2. Crystallization

Protein crystallizations were set up using sitting-drop vapour diffusion in 200 and 300 nl drops at 298 K following previously published protocols (Walter *et al.*, 2005 ▸). Briefly, 40 µl crystallization solution was transferred to the solution reservoir subwell of a Swissci UV-transmissible Polymer 96-Well Triple Drop Plate (Molecular Dimensions) using a Hydra96 liquid-handling robot (Robbins Scientific), before 100 or 200 nl protein solution droplets were dispensed into 100 or 200 nl crystallization reservoir solution using a Mosquito liquid-handling robot (SPT Labtech) (Table 2 ▸). During initial crystallization screening, plates were prepared using 96-Well CrystalQuick X Plates (Greiner Bio-One). The plates were sealed and incubated at 298 K within a RockImager 1000 automated crystal-imaging system (Formulatrix). The plates were imaged according to an inverse Fibonacci spiral schedule and reviewed remotely.

### 2.3. Data collection and processing

Crystals were assessed by their morphology and absorbance of 280 nm light, and *in situ* X-ray diffraction data were collected at 293 K using a Dectris EIGER2 X 4M detector on beamline VMXi at Diamond Light Source (Sanchez-Weatherby*et al.*, 2019 ▸; Mikolajek *et al.*, 2023 ▸). Selected crystals were mounted using Dual Thickness MicroMounts and MicroLoops (MiTeGen), cryocooled in liquid nitrogen and X-ray diffraction data were collected at 100 K using a Dectris EIGER2 XE 16M detector on beamline I04 at Diamond Light Source (Table 3 ▸). All images were selected for data reduction in *XDS* (Kabsch, 2010 ▸) as implemented in *xia*2 (Winter, 2010 ▸), as part of the Diamond Light Source automated pipeline. Data merging was performed in *AIMLESS* (Evans & Murshudov, 2013 ▸) and 5% of the reflections were selected randomly to form an *R*_free_ set.

### 2.4. Structure solution and refinement

Structure solution and refinement were performed using the *CCP*4 suite of programs v8.0 (Agirre *et al.*, 2023 ▸). Molecular replacement for Fab D08 was performed in *Phaser* (McCoy *et al.*, 2007 ▸) using the protein chain H monomer of bovine ultralong antibody BLV5B8 (PDB entry 4k3e; Wang *et al.*, 2013 ▸) and the protein chain L monomer of bovine antibody B4HC-B13LC (PDB entry 6qn7; Ren *et al.*, 2019 ▸) as search models. MR placed one monomer in the asymmetric unit. This phasing solution was used with *ARP*/*wARP* (Morris *et al.*, 2002 ▸) for automated model building after rigid-body refinement in *REFMAC*5 (Murshudov *et al.*, 2011 ▸) in the resolution range 57.64–3.00 Å and refinement of atomic positions and *B* factors using the entire resolution range. Water molecules were added automatically to density above 3.0 r.m.s.d in the *F*_o_ − *F*_c_ map using *Coot* at the end of the refinement (Emsley *et al.*, 2010 ▸). Iterated model building was performed in *Coot* and led to a final *R* factor and *R*_free_ of 17.5% and 21.9%, respectively (Table 4 ▸). The model geometry was assessed using *MolProbity* (Williams *et al.*, 2018 ▸). Chain L of bovine antibody B4HC-B13LC (PDB entry 6qn7; Ren *et al.*, 2019 ▸) was used as a search model with the heavy chain of the D08 model for molecular replacement to solve the structure of Fab D08*. The solution of Fab BLV5B8* was achieved through molecular replacement using chain L of bovine antibody B4HC-B13LC (PDB entry 6qn7; Ren *et al.*, 2019 ▸) and chain H of bovine ultralong antibody BLV5B8 (PDB entry 4k3e; Wang *et al.*, 2013 ▸). Subsequent model refinement was performed using *REFMAC*5 (Murshudov *et al.*, 2011 ▸) (Table 4 ▸).

## 3. Results and discussion

**Figure 1:**
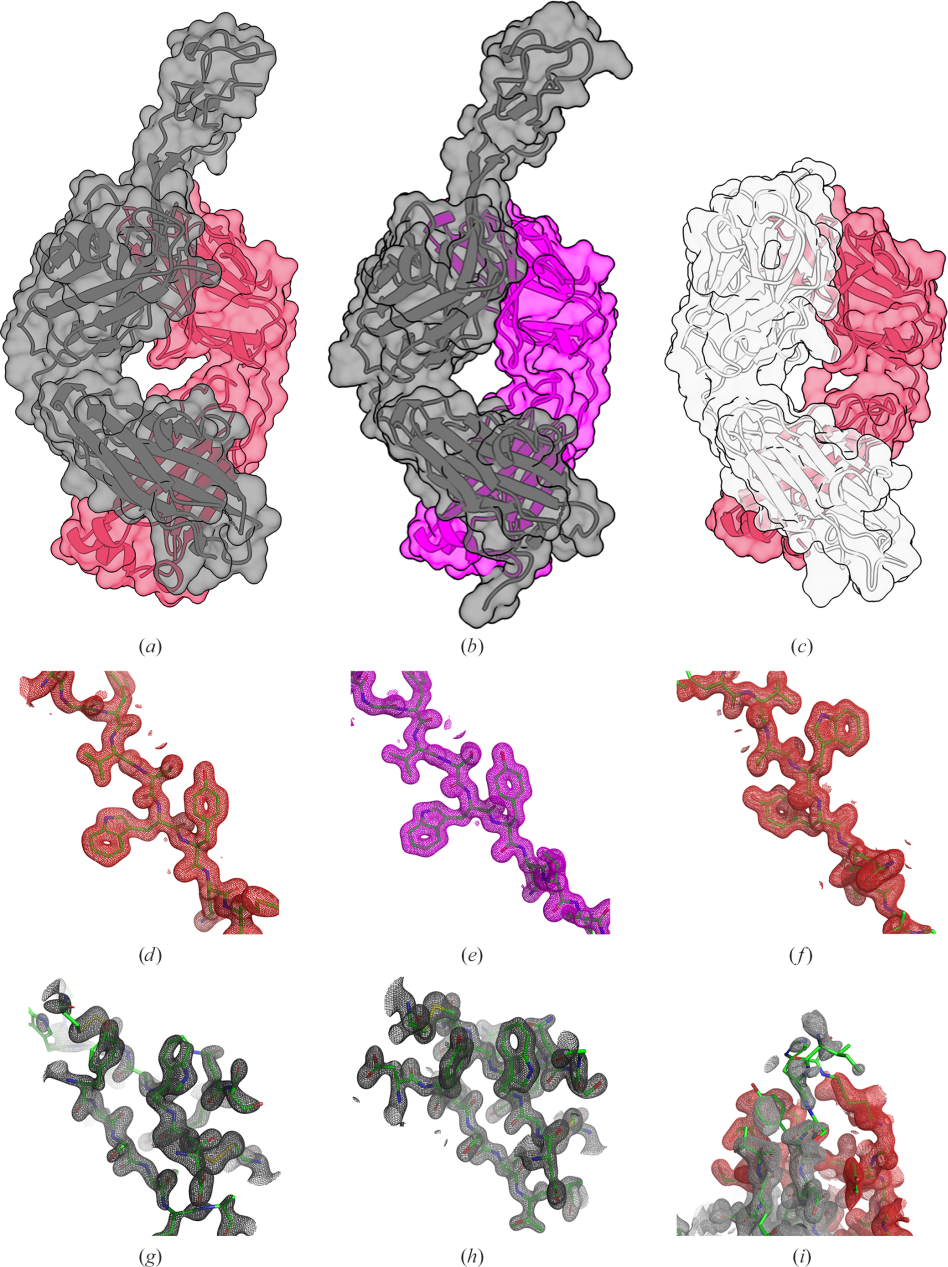
Overall crystal structures of ultralong Fabs D08 (*a*), D08* (*b*) and BLV5B8* (*c*), presented in cartoon representation with a semi-transparent surface overlay. Representative electron density is contoured in 1.0σ units and illustrated with mesh notation for the tryptophan at the core of the VL domain (*d*, *e*, *f*), the core of the knob mini-domain of Fabs D08 (*g*) and D08* (*h*) or the β-stalks of the ultralong loop stalk motif of BLV5B8* (*i*). The D08 heavy chain is coloured black and the D08 light chain is coloured red, while the BLV5B8 heavy chain is coloured white and the light chain is coloured magenta.

The ultralong antibody D08 was discovered as described in Section 2. Fabs were produced recombinantly for Fab D08, D08* (the D08 HC paired with the BLV5B8 LC) and BLV5B8* (the BLV5B8 HC paired with the D08 LC) (Table 1 ▸) and crystallized (Table 2 ▸). Structures were determined for all three molecules at between 1.45 and 1.59 Å resolution (Table 3 ▸). Continuous electron density was observed for both chains of all Fabs, with the following notable exceptions (Fig. 1 ▸): in Fab D08 Arg146 at the distal tip of the knob domain, the loop between residues 270 and 275 of the CH domain and the heavy-chain C-terminal eight residues, in Fab D08* the loop between residues 268 and 276 of the CH domain, and in Fab BLV5B8* the region between residues 102 and 144 of the CDR3H (Figs. 1 ▸*c* and 1 ▸*i*), the loops between residues 180 and 184, the region between residues 237 and 240, and the C-terminal eight residues of the CH domain, as well as the C-terminal serine of the light chain. The structures of both Fab D08 and D08* adopted the canonical ultralong antibody structure (Figs. 1 ▸*a* and 1 ▸*b*; Wang *et al.*, 2013 ▸; Stanfield *et al.*, 2016 ▸; Dong *et al.*, 2019 ▸) comprising a three-pass β-sheet knob domain, organized by a network of disulfide bonds, projected into the solvent by a β-ribbon stalk.

**Figure 2:**
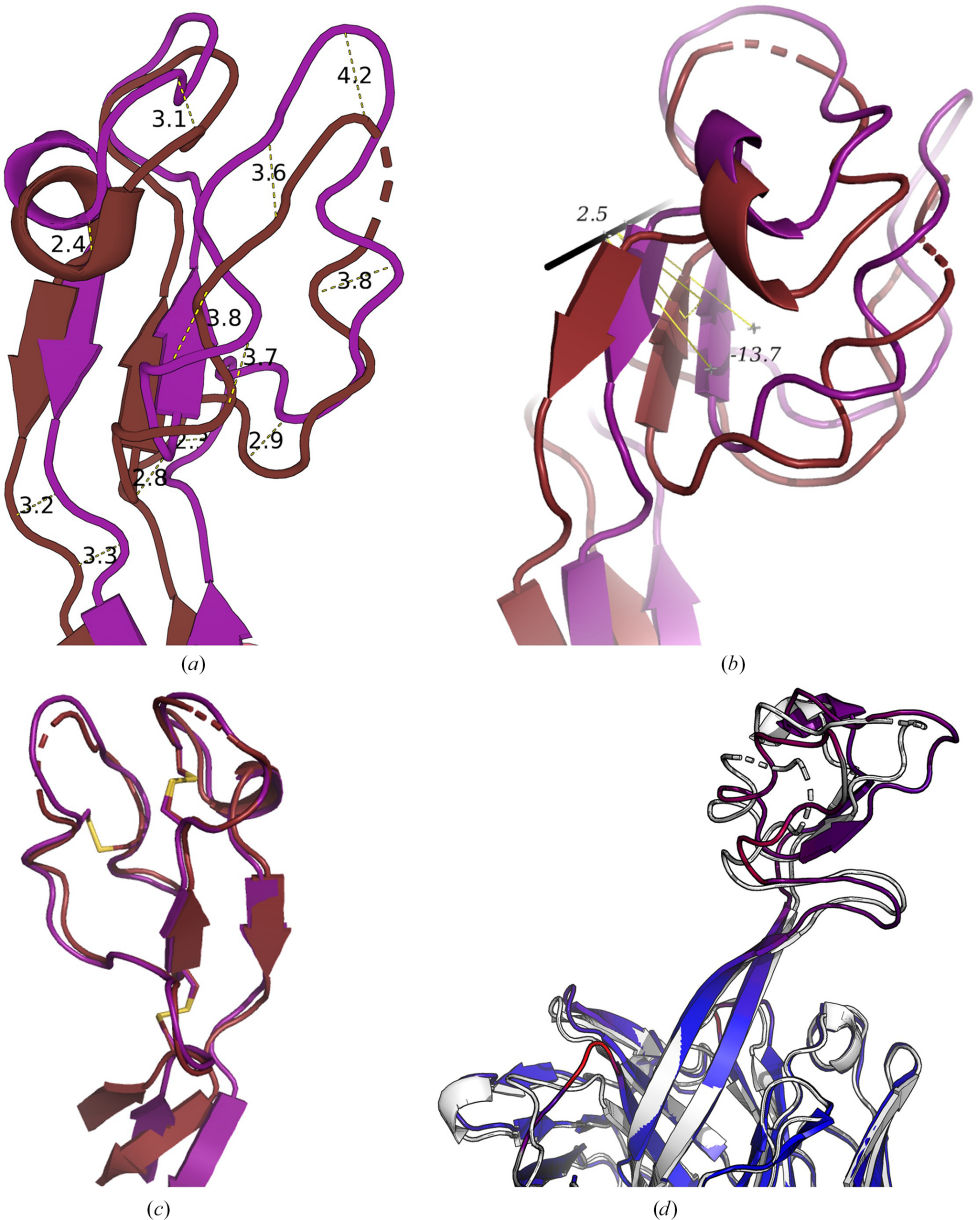
Comparison of the Fab D08 and D08* knob mini-domains. (*a*) Rotation and translation of D08 versus D08* knob mini-domains when the heavy chains are superimposed, with gross coordinate positional shifts indicated (*b*). (*c*) Superimposition of knob mini-domain cysteines with highlighted disulfide bridges. Knob mini-domains are coloured red for D08 and magenta for D08*. (*d*) Visualization of r.m.s.d. differences between superimposed Fab D08 (white) and D08* (coloured blue-to-red for r.m.s.d. differences in the range 0.05–6.93 Å). Rotational and positional shifts were calculated using the *RotationAxis PyMOL* script (Calvo, 2014 ▸). R.m.s.d. differences were visualized using the *ColorByRMSD PyMOL* script (Shandilya *et al.*, 2012 ▸).

The D08* knob mini-domain is displaced by up to 4.2 Å when superimposed on the D08 HC (Fig. 2 ▸*a*) due to a rotation of 13.72° and a translation of 2.47 Å of the ultralong loop (Fig. 2 ▸*b*) calculated using the *RotationAxisPyMOL* script (Calvo, 2014 ▸). Despite this displacement, if the cysteines of the knob mini-domains are superimposed, the knob and knob sub-loop main-chain architecture is preserved (Fig. 2 ▸*c*), supporting previous observations that the knob mini-domain acts as a rigid unit atop the β-stalk motif capable of binding antigen independently of the antibody scaffold (Svilenov *et al.*, 2021 ▸; Macpherson *et al.*, 2021 ▸; Huang *et al.*, 2023 ▸). Curious, however, is that several side chains present alternate rotamers between D08 and D08* (Figs. 1 ▸*a* and 1 ▸*b*).

**Figure 3:**
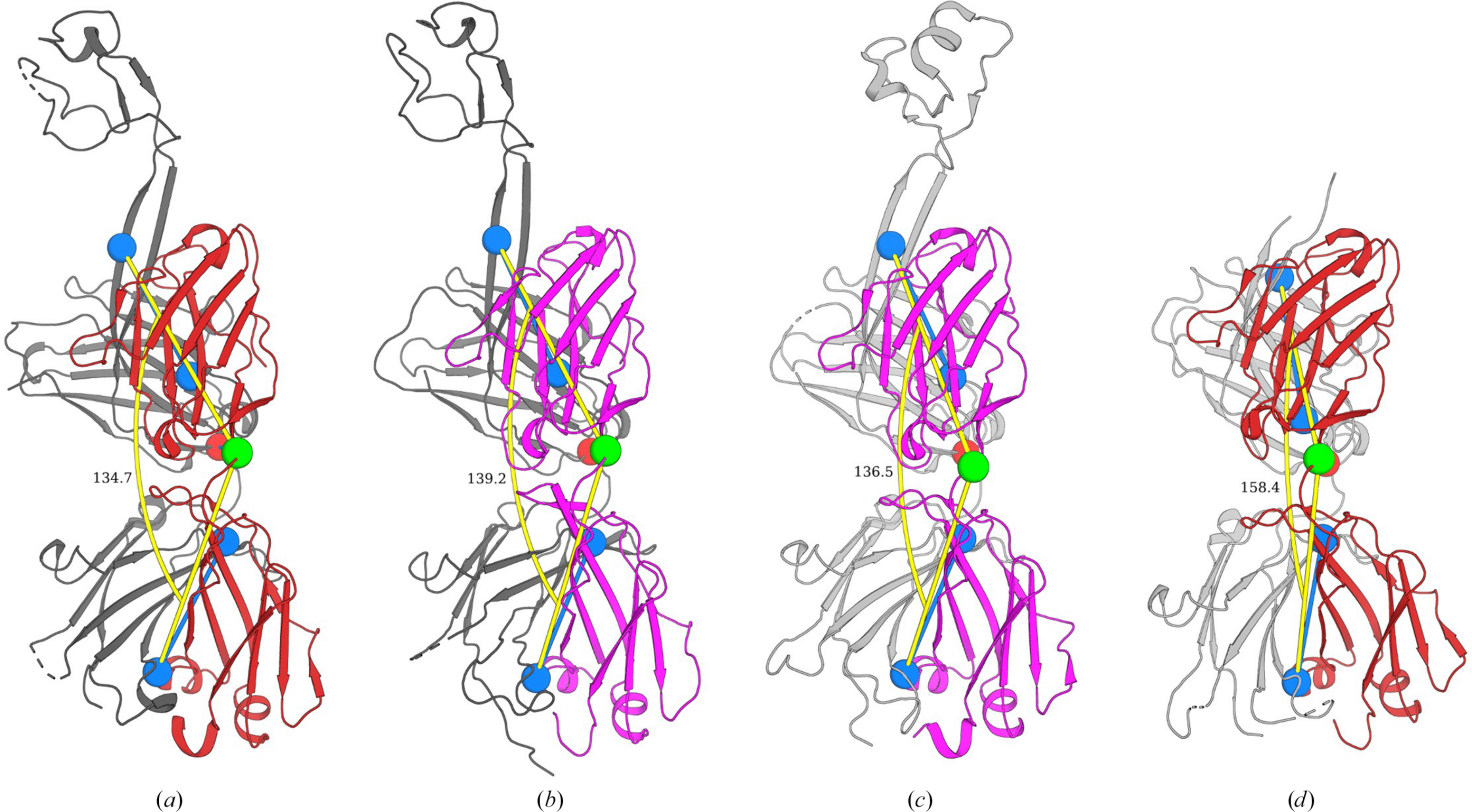
Elbow angles for Fabs D08 (*a*), D08* (*b*), BLV5B8 (*c*) and BLV5B8* (*d*) presented in cartoon representation. Chains are coloured as in Fig. 1 ▸: the BLV5B8 HC is in white, the D08 HC is in black, the BLV5B8 LC is in magenta and the D08 LC is in red. Blue dumbbells indicate psuedo-dyad rotation axes of each immunoglobulin domain with calculated hinge residues highlighted as green spheres (LC) or red spheres (HC). Elbow angles (Stanfield *et al.*, 2006 ▸) were visualized using the *PyMOL Elbow Angle* script (Sampson, 2014 ▸).

The displacement of the knob appears to be independent of the Fab elbow-bend angle, as the knob of BLV5B8 is rotated by 45.8° and translated by 0.9 Å relative to that of D08 (not illustrated in this manuscript), despite Fabs D08, D08* and BLV5B8 sharing similar elbow-bend angles of 134.7°, 139.2° and 136.5°, respectively (Figs. 3 ▸*a*–3 ▸*c*; Stanfield *et al.*, 2006 ▸). Furthermore, Fab BVL5B8* presented an elbow-bend angle of 158.4° (Fig. 3 ▸*d*) and crystallized in space group *C*121, whereas D08, D08* and BLV5B8 all crystallized in space group *P*2_1_2_1_2_1_ (Table 3 ▸). Interestingly, comparison of the elbow-bend angles of all deposited ultralong Fab structures to date revealed that those crystallized in space group *P*12_1_1 appear to demonstrate a bimodal distribution of accessible elbow angles (*n* = 4); however, no significant correlation of elbow angles was observed between different space groups (Supplementary Fig. S3; space groups containing fewer than four deposited structures were omitted from the statistical testing), thus there is no evidence that elbow angle and crystal packing are related in ultralong Fab structures.

**Figure 4:**
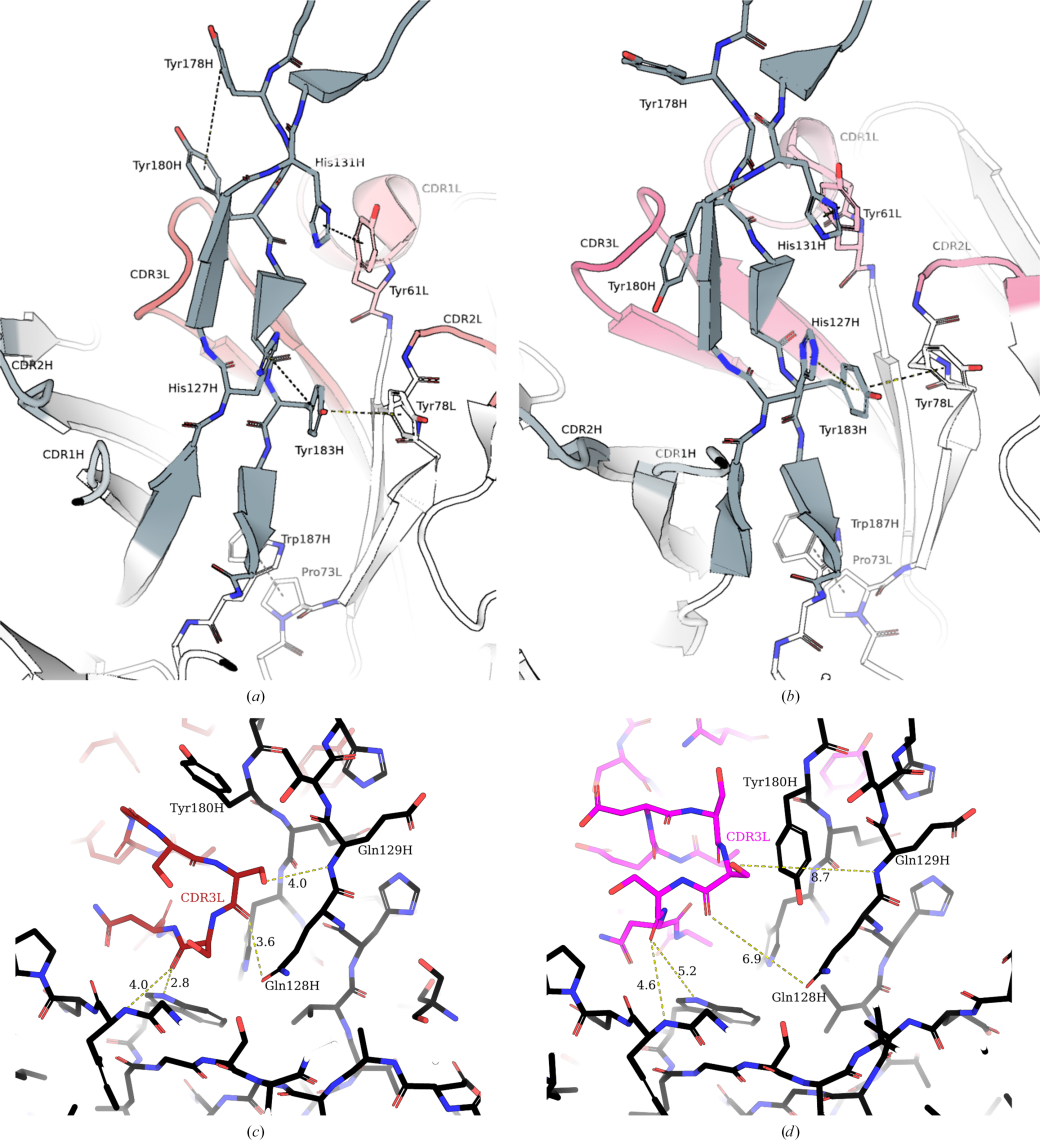
Molecular interactions supporting the ultralong stalk motif in ultralong Fabs (*a*, *c*) D08 and (*b*, *d*) D08* from side (*a*, *b*) and top-down (*c*, *d*) views. Complementarity-determining regions are coloured according to Fig. 1 ▸; the D08 heavy chain is in black, the D08 light chain is in red and the BLV5B8 light chain is in magenta, with increasing saturation from CDR1 to CDR3. For clarity, the field of view is focused on the ultralong CDR3H stalk motifs and immediate residues. π-Stacking interactions between aromatics are represented with dashed lines.

This displacement of the D08* knob mini-domain may be explained by examination of the molecular interactions supporting the ultralong stalk region. The D08 β-ribbon stalk is supported by a network of aromatic π-stacking interactions involving both the heavy and light chains, which tightly pack the CDR3L against the ascending and descending β-strands of the ultralong CDR3H (Fig. 4 ▸). Ascending from the base of the stalk towards the knob mini-domain, Trp187H stacks with Pro73L, Tyr183H is stacked between His127H and Tyr78L, and His131H stacks with Tyr61L (within CDR1L) (Figs. 4 ▸*a* and 4 ▸*b*). The D08 light-chain CDR3L adopts a tight turn conformation (Fig. 4 ▸*c*), allowing close packing against the side chain of Gln128H and the Glu129H backbone of the ascending strand of the D08 CDR3H, allowing π-stacking of Tyr180H with Tyr178H on the descending strand of the D08 CDR3H (Fig. 4 ▸*c*). Conversely, the BLV5B8 light-chain CDR3L adopts a β-turn structure that is too large to pack against the ascending strand of the D08 CDR3H, thereby allowing Tyr180H to pack between the BLV5B8 CDR3L and the D08 CDR3H (Fig. 4 ▸*d*). The close packing of Tyr180H to Gln128H and Glu129H, instead of the CDR3L, disrupts the Tyr180H–Tyr178H interaction of D08 and introduces a twist to the β-strand motif in D08*. The D08 and D08* knobs present a 1–4, 2–3, 5–6 network of disulfide bonds, which has only been reported in one other ultralong antibody structure (PDB entry 8edf; Huang *et al.*, 2023 ▸). Finally, the D08 CDR1H is also repositioned (Fig. 2 ▸*d*), although this would be unlikely to affect antigen recognition in an ultralong antibody system.

**Figure 5:**
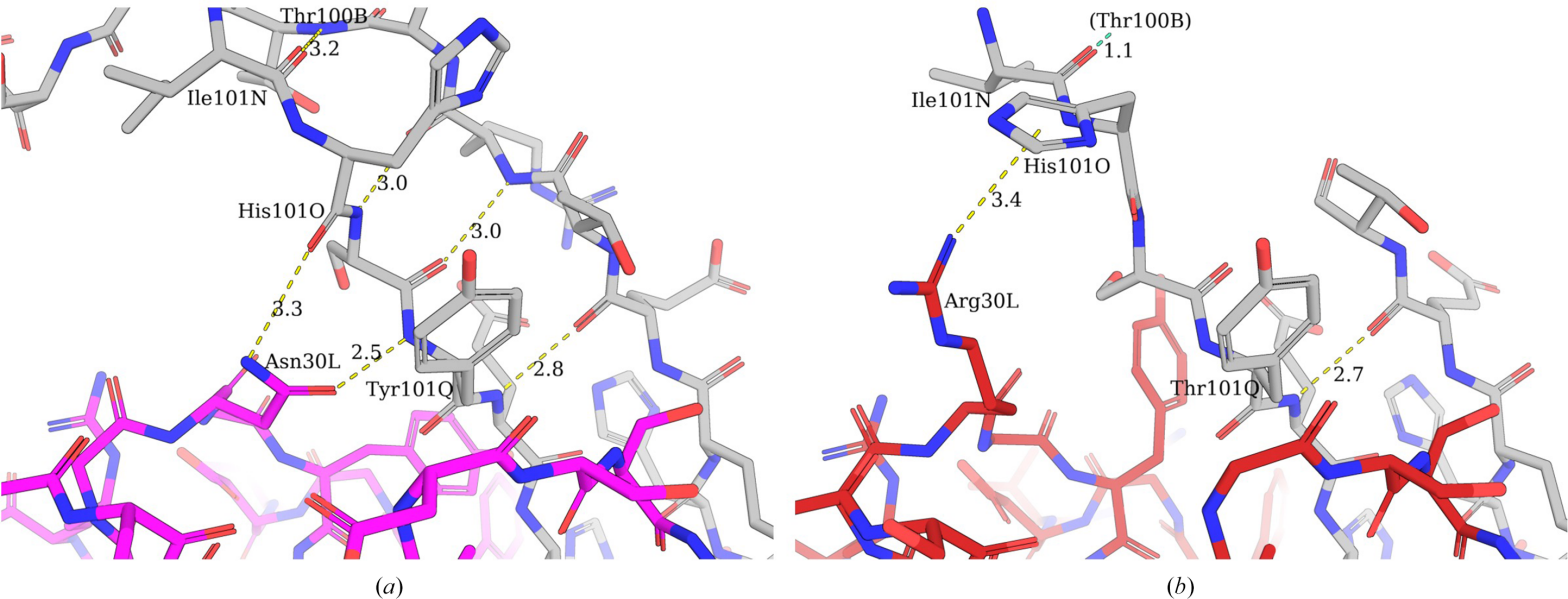
Interactions of the light chain with the descending strand of the BLV5B8 ultralong CDR3H. Descending β-strands of Fabs BLV5B8 (*a*) (Wang *et al.*, 2013 ▸) and BLV5B8* (*b*) are aligned and shown in stick notation; the BLV5B8 HC is in grey, the BLV5B8 LC is in magenta and the D08 LC is in red. The D08 and BLV5B8 ultralong CDR3H loops extend beyond the top of the figure, whilst the variable domains extend below. Hydrogen bonds of interest are indicated with yellow dashed lines. In (*b*), the cyan dashed lines indicate the proximity of the Ile101N (BLV5B8*) carboxyl group to the amine group of the aligned Thr100B (BLV5B8). Note that the BLV5B8 heavy chain is numbered using the numbering scheme of Chothia *et al.* (1986 ▸).

Intriguingly, the BLV5B8* ultralong CDR3H was not visible in the electron-density map (Fig. 1 ▸*i*), consistent with a greater degree of flexibility when paired with the D08 light chain, whereas this region is visible in the published crystal structure of BLV5B8 (PDB entry 4k3e; Wang *et al.*, 2013 ▸). One explanation for this flexibility may be the loss of a pair of hydrogen bonds between the side chain of Asn30L of the BLV5B8 LC, and the backbone amino group of Tyr101Q and the carbonyl group of His101O of the BLV5B5 HC (Fig. 5 ▸*a*). In the D08 LC, this asparagine is substituted by an arginine orientated away from the HC (Fig. 5 ▸*b*). Furthermore, the guanidine group at the distal end of the arginine is positioned to interact with the flipped pyrrole of His101O, which may also contribute to perturbing the ultralong loop of BLV5B8. Taken together, the observed descending strand of BLV5B8* HC projects closer to the coordinates of the ascending strand of BLV5B8; Ile101NH is shifted such that if superimposed with the BLV5B8 Fab (aligned at the base of the stalks), it would cause a steric clash with Thr100B of the ascending strand (Fig. 5 ▸*b*; cyan dashes).

Inspection of the crystal packing of BLV5B8* shows a larger cavity at the ultralong loop than observed in the D08, D08* or BLV5B8 crystals. BLV5B8* crystallized in space group *C*121 (whereas D08, D08* and BLV5B8 crystallized in space group *P*2_1_2_1_2_1_); however, other *C*121 ultralong structures presented an ordered knob mini-domain within a more compact cavity than present in the BLV5V8* crystal (Supplementary Fig. S4). This suggests that the observed ultralong position may be due to crystal-packing constraints, that the ultralong CDR3H is highly flexible and that the light chain may confer little-to-no structural input. In support of this conclusion, *PISA* analysis (Krissinel & Henrick, 2007 ▸) shows that the D08 and D08* knob mini-domains are heavily involved in crystalline interfaces (Supplementary Fig. S5).

Taken in isolation, the results presented here might suggest that residues of both the HC and LC immediately adjacent to the ultralong stalk are able to alter the fine positioning of the knob mini-domain; however, this effect cannot be deconvoluted from the effect of crystal-packing constraints. Indeed, the ultralong CDR3H makes extensive crystallographic interfaces, indicating that the observed positioning of ultralong CDR3H knob mini-domains may be more dependent on crystalline packing than antibody composition. Considering that the knob mini-domain makes the sole contact with the antigen (Wang *et al.*, 2013 ▸; Svilenov *et al.*, 2021 ▸; Macpherson *et al.*, 2021 ▸; Huang *et al.*, 2023 ▸), it appears that the selection of the light chain is irrelevant for maintenance of the ultralong CDR3H structure. In absence of a known antigen against which to test these chain-exchanged variants, it remains unknown whether the light-chain selection could affect antibody performance in terms of kinetics or specificity, although this would intuitively seem unlikely.

## Supplementary Material

PDB reference: BLV5B8*, 8p6h

PDB reference: D08*, 8p2t

PDB reference: D08, 8bs8

Supplementary Figures. DOI: 10.1107/S2053230X2400606X/rl5199sup1.pdf

## Figures and Tables

**Table 1:** Macromolecule-production information

Source organism	
Heavy chain	*Bos taurus*
Light chain	*Bos taurus*
DNA source	Sorted single B cells
Cloning vector	
Heavy chain	pOPINBOVH
Light chain	pOPINBOVL
Expression vector	
Heavy chain	pOPINBOVH
Light chain	pOPINBOVL
Expression host	Expi293
Complete amino-acid sequences of Fab D08	
Heavy chain	MGILPSPGMPALLSLVSLLSVLLMGCVAQVQLRESGPSLVKPSQTLSLTCTASGSSLSDEAVGWVRQAPGKSLEWLGSIDTGGNTGYNPNLKTRLSITKDNSKSQVSLSMSSVTPEDSATYFCATVHQETHQTCPDGYNSGDDCGRGNWDCGTLDCWRCDWGGFCRASTDYRSVTATYTYEWYIDTWGQGLLVTVSSASTTAPKVYPLSSCCGDKSSSTVTLGCLVSSYMPEPVTVTWNSGALKSGVHTFPAVLQSSGLYSLSSMVTVPGSTSGTQTFTCNVAHPASSTKVDKAVDPRCGKHHHHHH
Light chain	MGILPSPGMPALLSLVSLLSVLLMGCVAQGVLTQPSSVSGSLGQRVSITCSGSSSNVGRGYVSWYQMTPGSAPRTLIYGDTNRASGVPDRFSASRSGNTATLTISSLQAEDEADYFCASAEGSSSNAVFGSGTTLTVLGQPKSPPSVTLFPPSTEELNGNKATLVCLISDFYPGSVTVVWKADGSTITRNVETTRASKQSNSKYAASSYLSLTSSDWKSKGSYSCEVTHEGSTVTKTVKPSECS
Complete amino-acid sequences of Fab D08*	
Heavy chain	MGILPSPGMPALLSLVSLLSVLLMGCVAQVQLRESGPSLVKPSQTLSLTCTASGSSLSDEAVGWVRQAPGKSLEWLGSIDTGGNTGYNPNLKTRLSITKDNSKSQVSLSMSSVTPEDSATYFCATVHQETHQTCPDGYNSGDDCGRGNWDCGTLDCWRCDWGGFCRASTDYRSVTATYTYEWYIDTWGQGLLVTVSSASTTAPKVYPLSSCCGDKSSSTVTLGCLVSSYMPEPVTVTWNSGALKSGVHTFPAVLQSSGLYSLSSMVTVPGSTSGTQTFTCNVAHPASSTKVDKAVDPRCGKHHHHHH
Light chain	MGILPSPGMPALLSLVSLLSVLLMGCVAQPSSVSGSLGQRVSITCSGSSSNVGNGYVSWYQLIPGSAPRTLIYGDTSRASGVPDRFSGSRSGNTATLTISSLQAEDEADYFCASAEDSSSNAVFGSGTTLTVLGQPKSPPSVTLFPPSTEELNGNKATLVCLISDFYPGSVTVVWKADGSTITRNVETTRASKQSNSKYAASSYLSLTSSDWKSKGSYSCEVTHEGSTVTKTVKPSECS
Complete amino-acid sequences of Fab BLV5B8*	
Heavy chain	MGILPSPGMPALLSLVSLLSVLLMGCVAQVQLRESGPSLVQPSQTLSLTCTASGFSLSDKAVGWVRQAPGKALEWLGSIDTGGSTGYNPGLKSRLSITKDNSKSQVSLSVSSVTTEDSATYYCTTVHQETRKTCSDGYIAVDSCGRGQSDGCVNDCNSCYYGWRNCRRQPAIHSYEFHVDAWGRGLLVTVSSASTTAPKVYPLSSCCGDKSSSTVTLGCLVSSYMPEPVTVTWNSGALKSGVHTFPAVLQSSGLYSLSSMVTVPGSTSGTQTFTCNVAHPASSTKVDKAVDPRCGKHHHHHH
Light chain	MGILPSPGMPALLSLVSLLSVLLMGCVAQGVLTQPSSVSGSLGQRVSITCSGSSSNVGRGYVSWYQMTPGSAPRTLIYGDTNRASGVPDRFSASRSGNTATLTISSLQAEDEADYFCASAEGSSSNAVFGSGTTLTVLGQPKSPPSVTLFPPSTEELNGNKATLVCLISDFYPGSVTVVWKADGSTITRNVETTRASKQSNSKYAASSYLSLTSSDWKSKGSYSCEVTHEGSTVTKTVKPSECS

**Table 2:** Crystallization

Fragment antibody	D08	D08*	BLV5B8*
Method	Vapour diffusion, sitting drop		
Plate type	Swissci UV-transmissible Polymer 96-Well Triple Drop Plates (Molecular Dimensions)		
Temperature (K)	298		
Protein concentration (mg ml^−1^)	23.1	26.0	24.4
Buffer composition of protein solution	150 m*M* NaCl, 150 m*M* Tris–HCl pH 7.50		
Composition of reservoir solution	0.1 *M* Tris (base), 0.1 *M* Bicine, 12%(*v*/*v*) PEG 500 MME, 6%(*v*/*v*) PEG 20 000, 0.15 *M* sodium chloride, 0.09 *M* sodium nitrate, 0.09 *M* sodium phosphate dibasic, 0.09 *M* ammonium sulfate	0.1 *M* sodium HEPES, 0.1 *M* MOPS pH 7.50,15%(*v*/*v*) glycerol, 7.5%(*v*/*v*) PEG 4000, 0.09 *M* sodium fluoride, 0.09 *M* sodium bromide, 0.09 *M* sodium iodide	0.1 *M* sodium HEPES, 0.1 *M* MOPS pH 7.50, 12%(*v*/*v*) PEG 550 MME, 6%(*v*/*v*) PEG 20 000, 0.024 *M*D-glucose, 0.024 *M*D-mannose, 0.024 *M*D-galactose, 0.024 *M*L-fructose, 0.024 *M*D-xylose, 0.024 *MN*-acetyl-D-glucosamine
Volume and ratio of drop	200 nl (1:1 protein:reservoir solution)	300 nl (2:1 protein:reservoir solution)	300 nl (1:2 protein:reservoir solution)
Volume of reservoir (µl)	40	40	40

**Table 3:** Data collection and processing Values in parentheses are for the outer shell.

Fragment antibody	D08	D08*	BLV5B8*
Diffraction source	Beamline I04, Diamond Light Source		
Wavelength (Å)	0.9795		
Temperature (K)	100		
Detector	Dectris EIGER X 16M pixel		
Rotation range per image (°)	0.1	0.1	0.1
Total rotation range (°)	180.0	180.0	360.0
Exposure time per image (s)	0.008	0.008	0.008
Space group	*P*2_1_2_1_2_1_	*P*2_1_2_1_2_1_	*C*121
*a*, *b*, *c* (Å)	63.5, 71.1, 98.4	65.0, 70.9, 96.0	161.2, 49.0, 60.4
α, β, γ (°)	90.0, 90.0, 90.0	90.0, 90.0, 90.0	90.0, 90.9, 90.0
Resolution range (Å)	57.64–1.59 (1.62–1.59)	39.74–1.45 (1.48–1.45)	80.57–1.46 (1.50–1.46)
Total No. of reflections	408647 (20025)	526665 (22235)	539903 (23229)
No. of unique reflections	60540 (3079)	79276 (3919)	82095 (5807)
Completeness (%)	100.0 (100.0)	100.0 (100.0)	100.0 (100.0)
Multiplicity	6.7 (6.8)	6.6 (5.7)	6.6 (5.7)
〈*I*/σ(*I*)〉	14.0 (1.3)	10.7 (0.5)	12.0 (0.4)
*R*_r.i.m._	0.063 (1.416)	0.076 (1.946)	0.076 (2.019)
*R*_p.i.m._	0.024 (0.710)	0.029 (0.806)	0.029 (0.847)
Overall *B* factor from Wilson plot (Å^2^)	27	24	24

**Table 4:** Structure refinement Values in parentheses are for the outer shell.

Fragment antibody	D08	D08*	BLV5B8*
Resolution range (Å)	57.64–1.59 (1.63–1.59)	39.74–1.45 (1.48–1.45)	80.57–1.46 (1.50–1.46)
Completeness (%)	100.0 (100.0)	100.0 (100.0)	100.0 (100.0)
No. of reflections, working set	57461 (4157)	75241 (5487)	82095 (5807)
No. of reflections, test set	3079 (222)	3958 (260)	3984 (263)
Final *R*_cryst_	0.175 (0.297)	0.180 (0.346)	0.222 (0.384)†
Final *R*_free_	0.219 (0.320)	0.219 (0.373)	0.261 (0.399)†
No. of non-H atoms			
Protein	3490	3584	3171
Ligand	8	9	0
Water	590	539	290
Total	4088	4133	3461
R.m.s. deviations			
Bond lengths (Å)	0.0080	0.0089	0.0084
Angles (°)	1.478	1.533	1.528
Average *B* factors (Å^2^)			
Protein	31	27	33
Ligand	52	39	NA
Water	42	40	39
Ramachandran plot			
Most favoured (%)	455 [95.99%]	457 [95.81%]	404 [95.28%]
Allowed (%)	15 [3.16%]	17 [3.56%]	14 [3.30%]

† The electron-density map for this model is missing a tract of 42 amino acids at the ultralong CDR3H, which contributes to the higher *R* values.

## Data Availability

All data created during this research is openly available from the Protein Data Bank at the provided accession numbers. This study includes re-analysis of existing data, which is openly available at locations cited in the references section.
