# Supplementary material for: The impact of exchanging the light and heavy chains on the structures of bovine ultralong antibodies
Source: Acta Crystallogr F Struct Biol Commun. 2024 Jul 1;80(Pt 7):154–63. doi: 10.1107/S2053230X2400606X (PMC11229553; doi:10.1107/S2053230X2400606X)
Supplement: Supplementary file 1 [file f-80-00154-sup1.pdf]

STRUCTURAL BIOLOGY COMMUNICATIONS

Volume 80 (2024)

Supporting information for article:

The impact of exchanging the light and heavy chains on the structures of bovine ultralong antibodies

John D. Clarke, Alice Douangamath, Halina Mikolajek, Marie Bonnet-Di Placido, Jingshan Ren, Elizabeth E. Fry, Dave I. Stuart, John A. Hammond and Raymond J. Owens

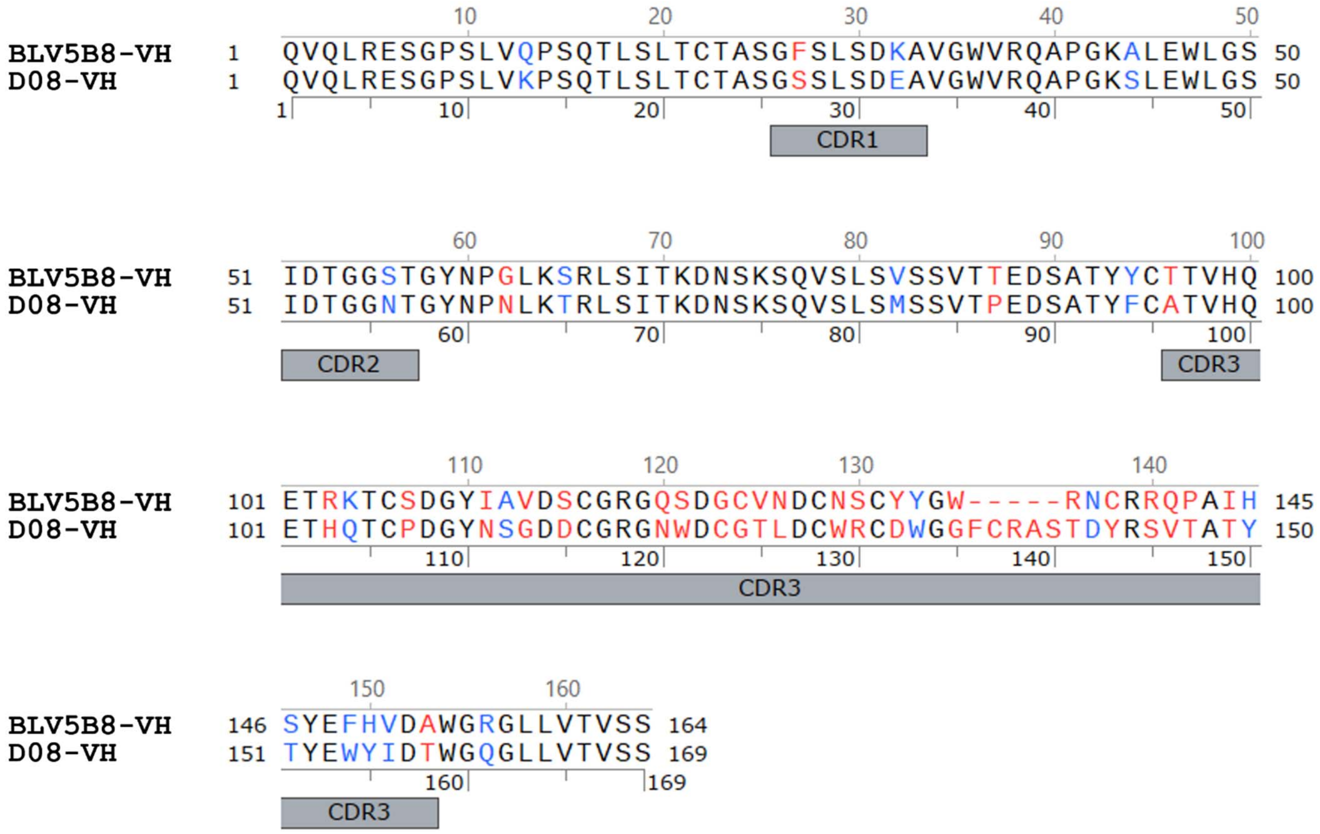

Figure S1 Multiple Sequence Alignment of the BLV5B8 and D08 heavy chain variable domains. Black indicates residue identity, blue indicates similarity, and red indicates differences. Positions of CDRs are marked.

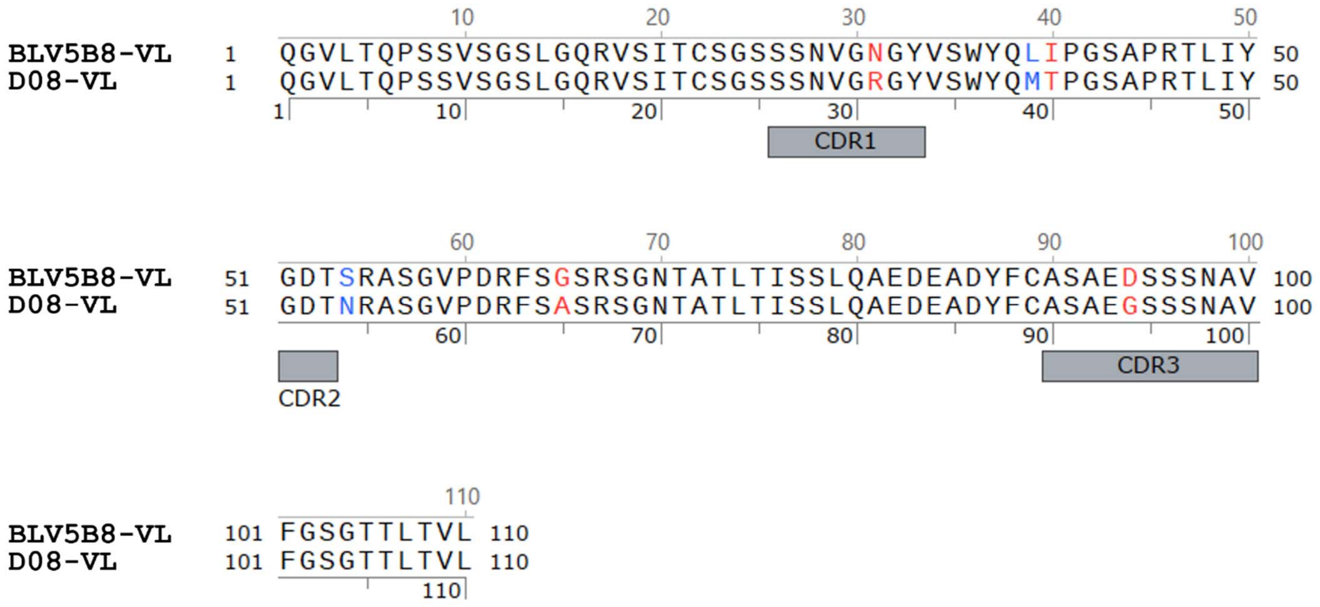

Figure S2 Multiple Sequence Alignment of the BLV5B8 and D08 light chain variable domains. Black indicates residue identity, blue indicates similarity, and red indicates differences. Positions of CDR's are marked.

S1. Description of gene segment recombinations, and shared identity between each heavy chain and light chain

The light chain of both antibodies D08, and BLV5B8 were produced by a V-J recombination involving gene segments IgLV1-47*01 and IgLJ4*01, and harbour a CDR3L of 11 residues. The D08 and BLV5B8 light chains share 94.29 % and 90.91 % identity over the variable domain, and CDR3L respectively.

The heavy chain of both antibodies D08, and BLV5B8 were produced by a V-D-J recombination involving gene segments IgHV1-7*02, IgHD8-2*01, and IgHJ2-4*01, and harbour a CDR3H of 65 and 54 residues respectively. The D08 and BLV5B8 heavy chains share 71.60 % and 42.62 % identity over the variable domain, and CDR3H respectively.

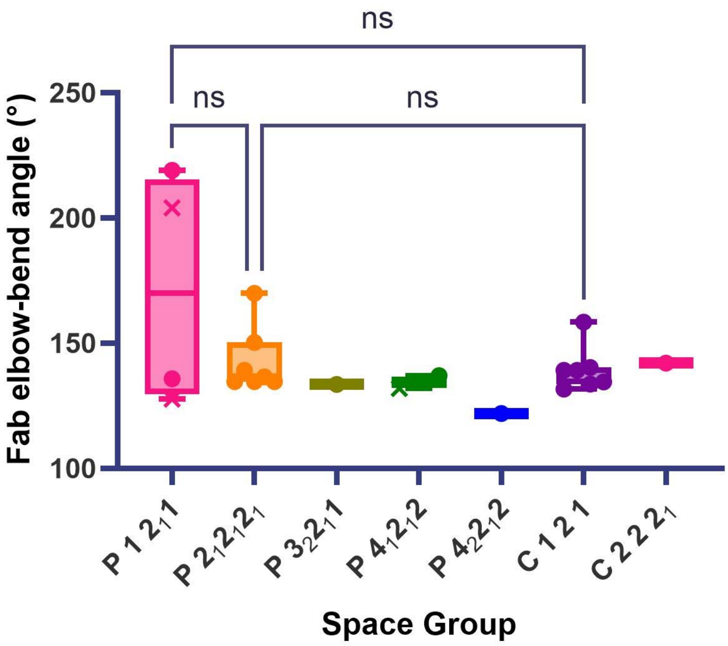

Figure S3 Comparison of elbow-bend angles (Stanfield *et al.*, 2006) and space groups for deposited ultralong Fab structures. Where significant additional contributions to the pseudo-twofold axes were identified, these points are plotted as a cross. Kruskal-Wallis test was performed (due to small sample sizes and non-gaussian distribution of elbow angles) with multiple comparisons between space groups comprising 4 or greater structures. P value for all comparisons was >0.9999.

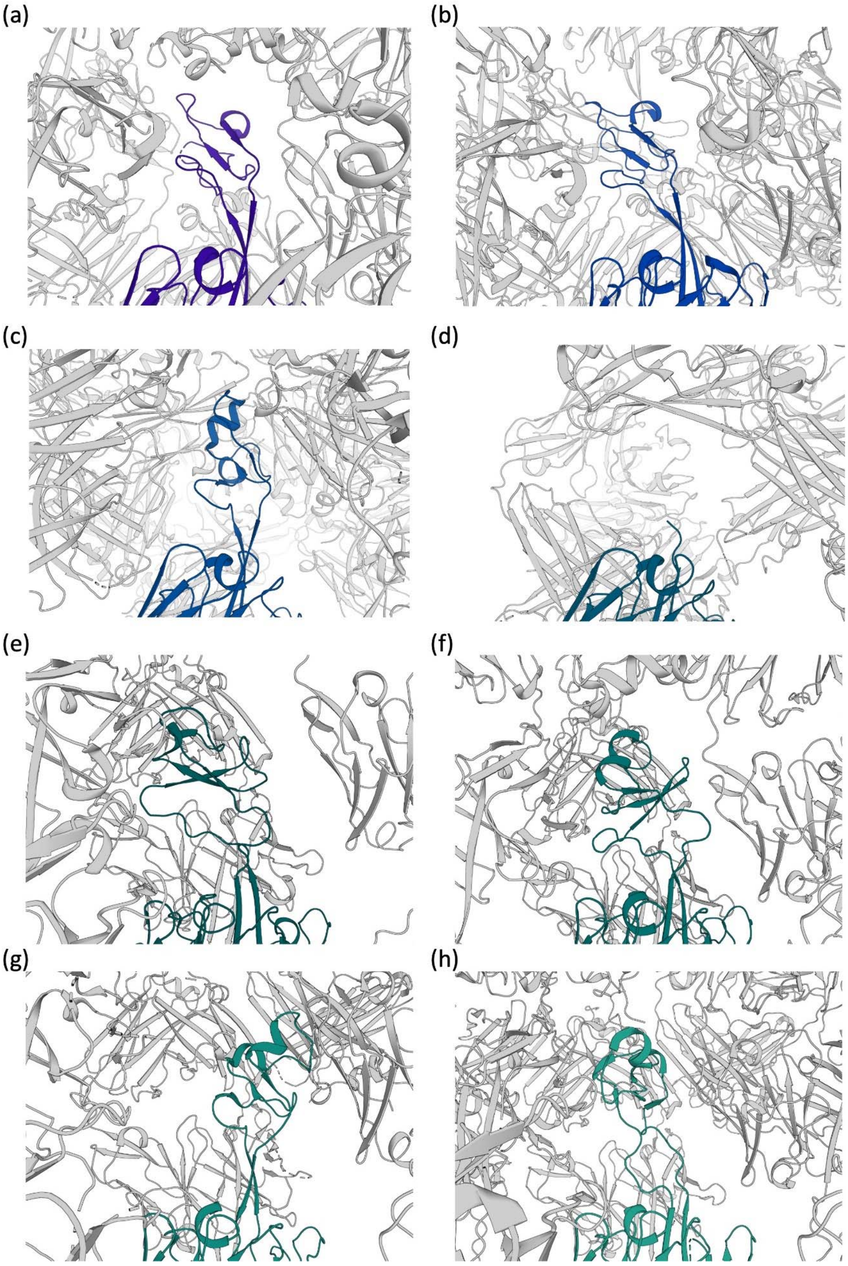

Figure S4 Crystalline packing of ultralong loops of antibodies (a) D08, (b) D08*, (c) BLV5B8 (PDB ID: 4K3E; Wang *et al.*, 2013), (d) BLV5B8*, (e) B11 (PDB ID: 5IHU; Stanfield *et al.*, 2016), (f) A01 (PDB ID: 5ILT; Stanfield *et al.*, 2016), (g) BOV-2 (PDB ID: 6E9G; Dong *et al.*, 2019), and (h) NC-Cow1 (PDB ID: 6OO0; Stanfield *et al.*, 2020). Crystals presented space groups $P\ 2_1\ 2_1\ 2_1$ (a-c) or $C\ 1\ 2\ 1$ (d-h).

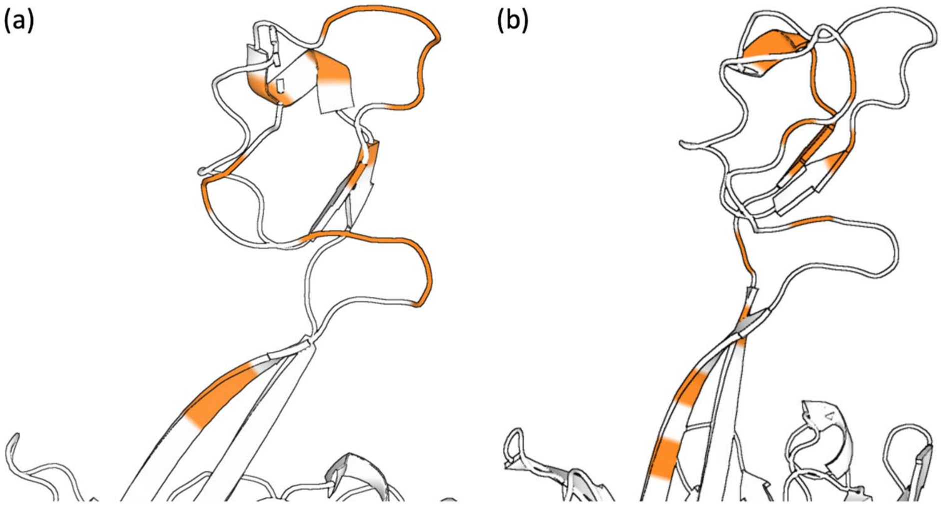

Figure S5 The ultralong knob mini-domain makes extensive crystalline contacts with symmetry mates. The knob mini-domains of (a) Fab D08, and (b) Fab D08*, with crystallographic interfacing residues coloured orange, as identified by PISA analysis (Krissinel & Henrick, 2007).
